# Infection control of COVID-19 in pediatric tertiary care hospitals: challenges and implications for future pandemics

**DOI:** 10.1186/s12887-022-03299-x

**Published:** 2022-04-26

**Authors:** Jonathan Remppis, Johannes Hilberath, Tina Ganzenmüller, Christoph Slavetinsky, Malte Kohns Vasconcelos, Marion Gnädig, Jan Liese, Siri Göpel, Peter Lang, Oliver Heinzel, Hanna Renk

**Affiliations:** 1 Department of Hematology and Oncology, University Children’s Hospital Tuebingen, Hoppe-Seyler-Straße 1, 72076 Tuebingen, Germany; 2 Institute for Medical Virology and Epidemiology, University Hospital Tuebingen, Tuebingen, Germany; 3 German Center for Infection Research (DZIF), Tuebingen, Germany; 4 Interfaculty Institute of Microbiology and Infection Medicine, University of Tuebingen, Tuebingen, Germany; 5 Pediatric Surgery and Urology, University Children´s Hospital Tuebingen, University of Tuebingen, Tuebingen, Germany; 6 Institute for Medical Microbiology and Hospital Hygiene, Heinrich Heine University Duesseldorf, Duesseldorf, Germany; 7 Institute of Medical Microbiology and Hygiene, University Hospital Tuebingen, Tuebingen, Germany; 8 Infectious Diseases, Department of Internal Medicine I, University Hospital Tuebingen, Tuebingen, Germany

## Abstract

**Background:**

More than 2 years into the COVID-19 pandemic, SARS-CoV-2 still impacts children’s health and the management of pediatric hospitals. However, it is unclear which hygiene and infection control measures are effective and useful for pediatric hospitals. Here, we report infection control measures implemented at a tertiary care children’s hospital. We evaluated frequency of SARS-CoV-2 detection in admitted patients, in-hospital transmission and infection related findings. Furthermore, we aimed to capture perspectives of health-care workers and caregivers on effectiveness and burden of infection control measures. Knowledge gained can inform management of the ongoing and future pandemics.

**Methods:**

We designed a retrospective observational study and survey at a pediatric tertiary care referral center. Local infection control measures and respective guidelines regarding COVID-19 were reviewed. Three thousand seven hundred sixteen children under 18 years were tested for SARS-CoV-2 at the University Children’s Hospital Tuebingen and data on SARS-CoV-2 transmission were retrieved from internal records. Two surveys were conducted among 219 staff members and 229 caregivers.

**Results:**

Local infection control measures comprised the formation of a task force, triage, protective hygiene measures and an adaptable SARS-CoV-2 test strategy. Between January 2020 and March 2021, SARS-CoV-2 infection was detected in 37 children presenting to our hospital, 21 of these were admitted. One hospital-acquired infection occurred. About 90% of health-care staff perceived the majority of measures as effective and appropriate. However, visitor restrictions and cancellation of scheduled treatments were perceived least effective by hospital staff and as a particular burden for patients and their caregivers. Visits at the pediatric emergency department significantly decreased during the pandemic. We drafted a pandemic action plan by ranking infection control measures according to local transmission stages.

**Conclusions:**

SARS-CoV-2 infection control measures implemented in our tertiary care children’s hospital were evaluated by health-care workers as mostly effective and appropriate. In particular, good communication, transparency of decision-making as well as universal masking and infection screening were assessed as successful measures of infection control management. Visitor restrictions and cancellation of routine appointments, in contrast, were perceived as a particular burden on patient care and should be avoided. An established pandemic action plan may guide children’s hospitals in the future.

**Supplementary Information:**

The online version contains supplementary material available at 10.1186/s12887-022-03299-x.

## Background

Severe acute respiratory syndrome coronavirus 2 (SARS-CoV-2) has caused the pandemic of coronavirus disease 2019 (COVID-19), challenging health-care systems worldwide. Children in general develop milder disease in comparison to adults [1]. Nevertheless, severe courses of disease occur, and children with underlying health conditions have been recognized as risk group for severe illness [2–4]. This poses considerable challenges for tertiary care children’s hospitals caring for a large proportion of at-risk patients. These hospitals need to provide treatment for children suffering from COVID-19, protect other patients and health-care staff from hospital-acquired infection, and maintain specialized in- and outpatient care for risk groups.

This report will summarize infection control measures undertaken in a tertiary care children’s hospital during the first year of the pandemic, when a vaccine against SARS-CoV-2 was not yet widely available. The aim is to evaluate frequency of SARS-CoV-2 detection in admitted patients, in-hospital transmission and infection related findings with local infection control measures in place. Additionally, perspectives of health-care workers and caregivers on effectiveness and burden of infection control measures will be captured. Knowledge gained will result in the development of a pandemic action plan that may guide children’s hospitals in managing the ongoing SARS-CoV-2 and future pandemics.

## Methods

### Hospital setting

The University Children’s Hospital Tuebingen is a large tertiary care facility in southern Germany with a total of 170 beds. Risk groups treated at our hospital include those receiving hematopoietic stem cell transplantation, solid organ transplant, cardiac surgery, as well as children with rheumatologic diseases, cystic fibrosis and those requiring home ventilatory support. The hospital comprises a pediatric intensive care unit (PICU) and a neonatal intensive care unit (NICU). It has an emergency department (ED) as well as several outpatient clinics for high-risk patients and serves as regional primary and secondary care referral center.

### Study period and data collection

Data were collected from January 2020 until March 2021. Local guidelines developed during the course of the pandemic were obtained from the documentation of the local Corona task force, chronologically ordered by their release date and systematically reviewed. Data on SARS-CoV-2 tests conducted for pediatric patients during that time were provided by the local virology department (Additional file 1). To evaluate the perception of infection control measures among hospital staff members, an electronic questionnaire was created using the free software LimeSurvey (http://www.limesurvey.org, Additional file 2). The survey comprised 13 questions and investigated the perceived effect and burden of infection control measures at the children’s hospital and the quality of health-care among chronically ill children during the pandemic. The online-survey was circulated in February 2021 among all staff of the children’s hospital using a mailing list with approximately 600 email addresses. The survey was closed after ten days. In a second survey conducted between October 2020 and March 2021, one caregiver of each child visiting the outpatient clinics or the pediatric ED was asked about the perceived impact of the COVID-19 pandemic on their child’s health. As the two surveys assessed separate aspects of infection control, it was not intended to directly compare their results. Both questionnaires were developed with support of the Institute of Occupational and Social Medicine and Health Services Research and the local Centre for Pediatric Clinical Studies at our hospital. The finalized surveys were validated for their internal consistency, content and construct validity. Both surveys were completely anonymous and response options were designed as multiple-choice format in the majority of questions. Case numbers of pediatric ED visits and hospital admissions during the study period were retrospectively retrieved from the hospital records in the centralized SAP® Patient Management solution system. The study was approved by the independent ethics committee at the University Hospital Tuebingen (Project Numbers 023/2021BO2 and 686/2020BO2) with a waiver of informed consent and was performed in accordance with the ethical principles of the Declaration of Helsinki.

### Statistical analyses

Different items of the questionnaire were evaluated by descriptive analysis. Data are presented as percentages of total possible ratings of a particular measure or action. Microsoft Excel (Microsoft Excel 2010 v14.0, Microsoft Corporation, Redmond, Washington, USA) and SPSS (IBM SPSS Statistics 26, IBM, Armonk, New York, USA) were used for data collection and analysis.

The weekly number of unscheduled ED visits in children under 18 years in the year 2020 were compared with the number of visits during the corresponding weeks in the 5-year period from 2015 to 2019. A Shapiro-Wilk’s test (*p* > 0.05) (Shapiro &Wil, 1965; Razali &Wah, 2011) and a visual inspection of their histograms, normal Q-Q plots and box plots showed that the number of ED visits was not normally distributed for both periods, with a skewness of 0.71 (standard error [SE] 0,33) and a kurtosis of 0.51 (SE 0.66) for the period before and a skewness of 1.53 (SE 0.33) and a kurtosis of 1.3 (SE 0.66) for the period after [5, 6]. Since data were not normally distributed, Wilcoxon test was used for the comparison of the two groups. A *p*-value < 0.05 was considered statistically significant.

## Results

### Infection prevention and control

Due to the reports about COVID-19 spreading in Wuhan, China, a local task force was formed at the University Hospital Tuebingen by mid-January 2020, comprising specialists for infectious diseases, hospital hygiene, virology and intensive care medicine as well as other clinical disciplines (including pediatrics) and hospital management. The task force created the first local guidelines by the end of January 2020. During the following months a range of local infection control measures were implemented. A subordinate pediatric task force team adapted the respective measures to the needs of the pediatric setting. Infection control measures are described in Table 1, including their time of implementation, aims, and barriers and enablers (Table 1).

**Table 1:** Infection control measures

Measure	Time of implemen-tation	Aim	Barriers and enablers	Description
**Formation of a local Corona Task Force**	January 2020	To ensure effective management and functioning of the hospital and to link strategic and operational leaders with each other.	**Enablers:** Early formation of an informal team of specialists, already planning ahead. **Barriers:** Meetings tied up human resources that were needed in patient care.	Mid-January 2020: First informal meeting took place that comprised specialists for infectious diseases, hospital hygiene and intensive care as well as virologists. This was followed by the nomination of a leading physician of the Corona Task Force by the hospital medical board. Meetings with all relevant hospital stakeholders were called daily to weekly.
**Guidelines and standard operating procedures by local Corona task force**	January 2020	To ensure a rapid and consistent implementation of infection control measures adapted to local conditions	Enablers: Local availability of an electronic platform for guidelines and standard operating procedures.	Electronic guidelines accessible by all hospital staff. Regular adaptation to current situation. Contents: Local infection control measures, test strategy, isolation criteria, management of COVID-19 patients, visitor rules, vaccination for staff, and others.
**Enhanced communication**	January 2020	To ensure easy access and adherence of health care staff to local guidelines	Enablers: Local availability of a mailing system accessed by all hospital staff	Communication of the local situation and decisions of the task force was assigned to one hospital quality manager. Email updates were circulated among all staff daily to weekly in the first months of the pandemic. Implementation of an email address for questions and suggestions of staff and an emergency phone number for urgent inquiries.
**Triage at hospital entry and isolation area in pediatric ED**	Mid-February 2020	To prevent transmission of SARS-CoV-2 from patients in waiting areas and outpatient departments. To facilitate early testing of patients and their caregivers	Enablers: Availability of a separate access to the pediatric ED. Barriers: Previously, admission control during daytime had never been implemented. Lack of staff for triage at all access points. A separation area in the pediatric ED did not exist.	Recruitment of new staff to control patients’ and visitors’ access to the hospital. Closure of all access points apart from the main entrance. Screening for symptoms compatible with COVID-19, measurement of body temperature. Suspected cases were directly sent to a separate waiting area or examination room in the pediatric ED.
**Visitor restrictions**	Mid-March 2020	To prevent transmission of SARS-CoV-2 from visitors to patients and hospital staff	–	Restriction of accompanying persons to one parent or caregiver per patient. Complete restriction of visitors.
**Cancellation of non-urgent treatments**	Mid-March to End of June 2020; End of December 2020	To prevent crowding, re-allocate consultation and ward rooms for potential COVID-19 patients and preserve medical staff to maintain routine-care	Barriers: Lack of infrastructure for telemedical care in most pediatric departments.	Cancellation or postponement of: consultations because of long-term symptoms, day case surgeries (e.g. inguinal hernias, metal removal after osteosynthesis), follow up visits, referrals for second opinion and others.
**Mandatory face masks for staff, parents and patients**	End of March 2020 (FFP2-masks from February 2021)	To prevent SARS-CoV-2 transmission between patients, caregivers and staff	Barriers: Massive shortage of medical face masks (FFP1 and FFP2) during the first months of the pandemic. Uncertainty about their future availability.	Mandatory FFP1 masks in the entire hospital for staff, accompanying persons and patients ≥6 years of age. Only exception: inside ward rooms. Later in the pandemic, respirators (FFP2 or N95) became the obligatory standard.
**Respiratory infection ward/Isolation area on regular ward**	End of March 2020 /End of June 2020	To prevent virus transmission from admitted children with suspected or confirmed COVID-19. To facilitate specific care for COVID-19 patients by assigned health care staff	Enablers: Availability of additional ward rooms and health care staff from pediatric surgery due to the cancellation of scheduled non-urgent operations. Rapid building of interior walls to separate areas within wards was possible. Barriers: No pre-existing infection or isolation ward. Negative-pressure rooms not existing.	Dedicated ward for suspected or confirmed pediatric COVID-19 cases with a capacity of ten beds. Later, the ward was closed and replaced by an isolation area on another ward with a flexible capacity of three to five beds. Additionally, a flexible isolation area of max. Four beds was available on the PICU. Both areas were separated by installing additional doors and walls.
**Outpatient fever clinic**	End of March to Mid-April 2020	To minimize the risk of virus transmission from children seeking medical care for mild respiratory illness	Enablers: Effective collaboration with local authorities and rescue services	Outpatient fever clinic for children with suspected COVID-19 at another place in town, away from the hospital.
**Acquisition of local tools for SARS-CoV-2 testing**	End of February 2020	To provide quick and reliable tests in sufficient quantity	Enablers: Supportive local virology laboratory	Local SARS-CoV-2 tests available from February 2020. Increase of the monthly test capacity by more than four-fold between March and June 2020. Point-of-care polymerase chain reaction (PCR) testing available by November 2020. A detailed overview over the SARS-CoV-2 diagnostic test procedures used, their time of implementation, turnaround time and test capacity is given in Additional File 1.
**Implementation and regular adaptation of the local SARS-CoV-2 test strategy**	End of February 2020	Early detection of SARS-CoV-2 infection in symptomatic and asymptomatic patients and visitors	Enablers: Availability and updates of nation-wide guidelines on SARS-CoV-2 testing by the German public health authorities Barriers: Insufficiency of laboratory resources and long turnaround times for SARS-CoV-2 PCR testing during the first months of the pandemic	Shift of the indication for SARS-CoV-2 testing: - Mid-February: symptomatic travelers from risk areas and contact persons - Early-March: Symptomatic travelers from risk areas, any patients with pneumonia - Early-April: all patients admitted with symptoms compatible with COVID-19. All patients undergoing HSCT - Early-May: Routine-screening for patient admitted from ED, admitted to the PICU, patients in need of intubation or sedation, high-risk patients Mid-November: Point-of-care (POC)-PCR testing for all admissions from the pediatric ED; SARS-CoV-2 antigen tests for caregivers (on admission) and screening of staff once weekly.

*Abbreviations: COVID-19* coronavirus disease 2019, *ED* emergency department, *SARS-CoV-2* severe acute respiratory syndrome coronavirus 2, *PICU* pediatric intensive care unit

#### Test strategy

From February 2020, capacities for local tests on SARS-CoV-2 were built up by our virology department (Additional file 1). The overall purpose of screening was pre-hospital detection of SARS-CoV-2 infected patients and early detection of cases among health-care workers in order to control in-hospital spread. With increasing test capacities and a concurrent increase of local infections, the test strategy needed continuous adaptation. In summary, the indication for SARS-CoV-2 testing shifted from symptomatic travelers and contact persons to any symptomatic patients and, later in the course, to routine screening of asymptomatic patients, parents and staff (Table 1). The number of tests performed per month rapidly increased to more than four-fold between March and June 2020. With point-of-care polymerase chain reaction (PCR) testing available by November 2020, patients could be kept in the pediatric ED until test results were obtained.

### Infection related findings

Between 29 February 2020 and 31 March 2021, a total of 6915 respiratory specimens from 3716 different patients aged under 18 years were collected for molecular SARS-CoV-2 detection tests. The median age of the children tested was 4.9 years (interquartile range 1.4;11.0); 37 (1,0%) had a positive result. Twenty one children were admitted to the hospital with a positive SARS-CoV-2 PCR test. Seventeen were diagnosed on admission and two prior to admission, all of these were immediately isolated. Details about demography, clinical course, treatment and outcome of the admitted patients have been published elsewhere [4]. In the two remaining previously unknown cases, in-hospital transmission of SARS-CoV-2 was considered possible and further investigated. One patient had a negative SARS-CoV-2 PCR on admission but was tested positive during a follow-up PCR test 8 days later because of unexplained, persistent fever. She was immediately isolated on the respiratory infection ward thereafter, but had been at a regular ward for 8 days. Parents and staff were obliged to wear surgical masks by that time. Contact tracing among staff members led to the detection of a single SARS-CoV-2 infection in a nurse who had assisted with inhalation of nebulized saline solution. The nurse remained asymptomatic, no other transmission to health-care staff, patients or parents was detected. The second patient was a child admitted via the trauma room due to severe burning. The rapid antigen test was negative, but SARS-CoV-2 PCR weakly positive, probably as a sign of subsided infection (a cycle threshold (Ct) value was not available at that time). Five health-care workers, wearing medical face masks but no respirators (which became obligatory in the trauma room after this event), had been in close contact with the child. They were quarantined, but contact tracing showed that none had been infected. During the study period we did neither observe nor confirm any nosocomial transmission of SARS-CoV-2 from an infected child to another patient.

### Non-infection related findings

#### Perception of infection control measures among hospital staff

**Fig. 1:**
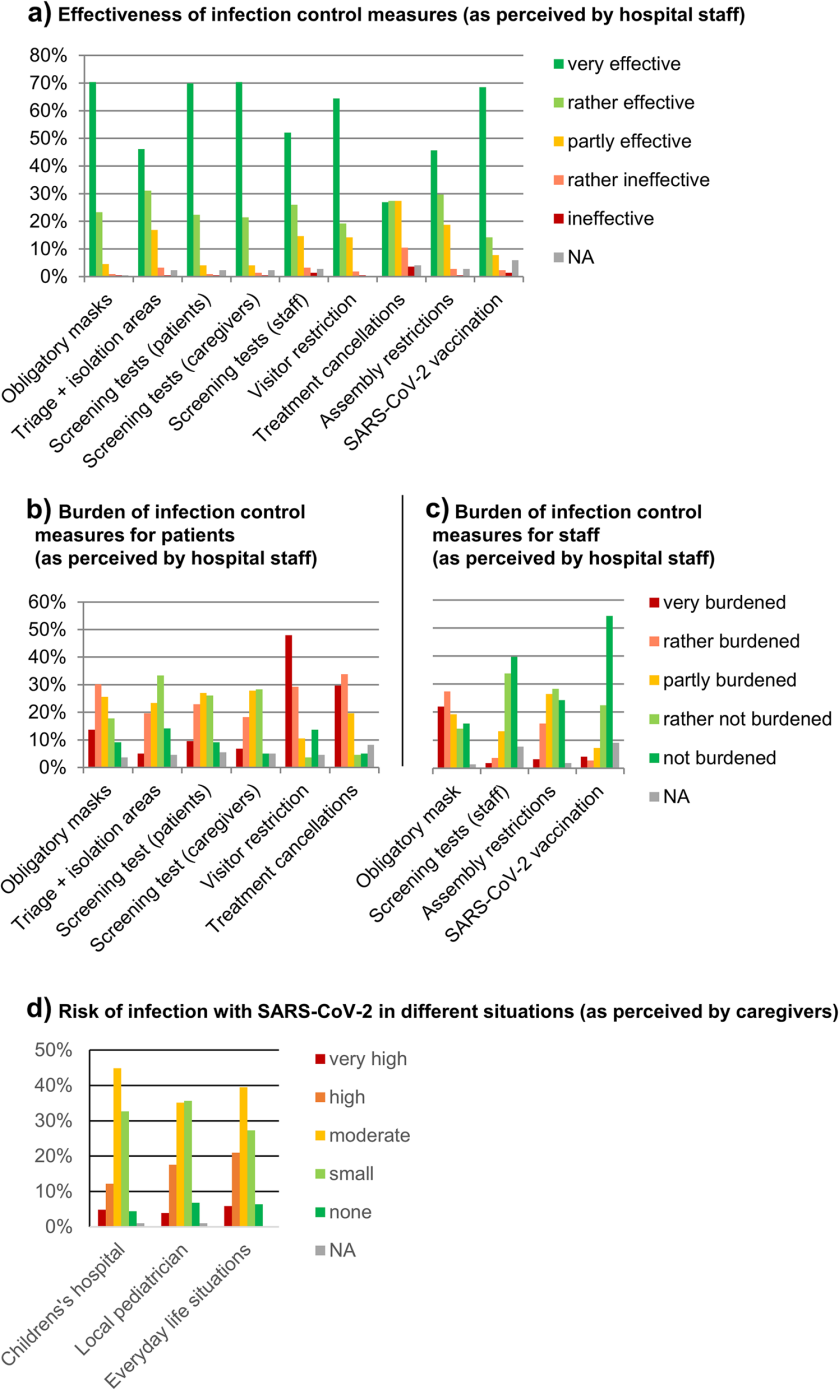
Effectiveness and burden of local infection control measures. **a** Effectiveness of infection control measures (as perceived by hospital staff). **b** Burden of infection control measures for patients (as perceived by hospital staff). **c** Burden of infection control measures for staff (as perceived by hospital staff). **d** Risk of infection with SARS-CoV-2 in different situations (as perceived by caregivers). **a** “How effective in the prevention of infections among staff and patients do you rate the following measures at your children’s hospital?”; **b** “In your perception, how burdened are patients and accompanying persons by the following measures at your children’s hospital?”; **c** “How burdened do you feel by the following measures at your children’s hospital?” **d** “How high do you estimate the risk, that your child(ren) get(s) infected with the coronavirus in the following situations: 1) everyday life situations (e.g. in the supermarket or on the playground) 2) during a visit at your GP or pediatrician 3) during a visit at a children’s hospital?” Abbreviations: SARS-CoV-2 severe acute respiratory syndrome 2, NA not available, reflecting the proportion of participants that did not answer the respective question

The online survey was completed by 219 staff members, including 112 (51%) nurses, 39 (18%) physicians and 61 (28%) participants of other professions (i.e. administration, laboratory, social work and others). At that time point, vaccination against SARS-CoV-2 had yet been received by 89 (41%) of the participants, 99 (45%) were willing to get themselves vaccinated, 21 (10%) were unwilling to get vaccinated. 179 (82%) felt well or rather well informed about the current status of the pandemic and infection control measures at the children’s hospital, 33 (15%) felt partly informed, and 6 (3%) rather badly informed; 163 (74%) felt well or rather well protected from COVID-19 by the infection control measures at the children’s hospital, 51 (23%) felt partly protected and 5 (2%) rather not protected. The perception of the effectiveness and burden of different infection control measures at the children’s hospital are presented in Fig. 1, answers stratified by profession are displayed in Additional file 3. In free commentaries, 24 participants remarked that the general obligation to wear respirators of a higher protection class (FFP2 or N95) in the hospital was a higher burden compared to surgical face masks.

Sixty-four (29%) participants estimated that the maintenance of care for chronically ill children had highly or rather suffered during the pandemic, 55 (25%) found it had partly suffered and 57 (26%) found it had not or rather not suffered. Complications caused by the infection control measures (e.g. delay of an urgently needed treatment) had been experienced frequently by 6 (3%) and sporadically by 69 (32%) participants, while 110 (50%) had not experienced any complications.

#### Perception of infection control measures among caregivers

Two hundred twenty-nine caregivers of patients who visited the emergency department or the outpatient clinic were offered participation in the additional questionnaire. Five persons refused to participate and 19 questionnaires were completed incorrectly. Two hundred five questionnaires were eligible for evaluation. 17.2% of all participants indicated that they considered the risk of SARS-CoV-2 infection during their visit at the children’s hospital to be very high or high, whereas 21.5 and 26.8% reported the risk as very high or high at their local pediatrician and in everyday life situations, respectively. 29.7 and 13.4% of the caregivers, respectively, stated that already arranged outpatient or inpatient appointments had to be cancelled or postponed.

#### Maintenance of pediatric emergency care

**Fig. 2:**
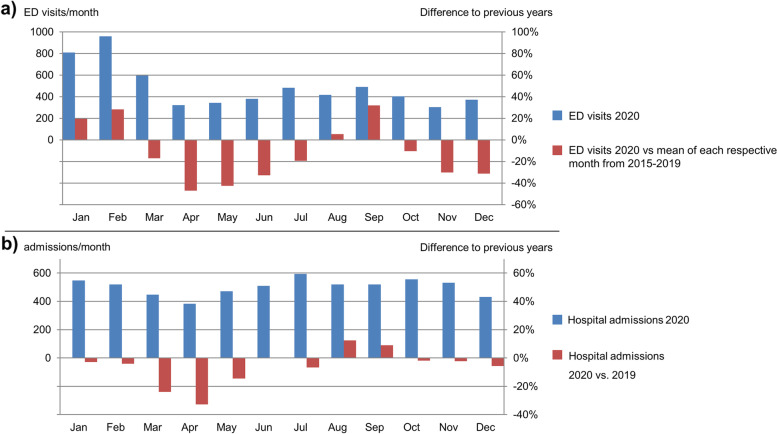
ED numbers and hospital admissions in 2020 in comparison to previous years. **a** ED numbers. Blue bars: numbers of unscheduled ED visits in patients < 18 years in 2020. Red bars: Difference between 2020 and the average of each respective month from 2015 to 2019 in percent; **b** Hospital admission. Blue bars: numbers of admissions to the children’s hospital in 2020. Red bars: Difference between 2020 and 2019 in percent. *Abbreviations: ED* emergency department

During the local spread of the pandemic, the number of ED visits and hospital admissions at our children’s hospital markedly decreased compared to previous years. While the number of ED visits remained low during the course of 2020 admission numbers rose again to the level of previous years by June 2020 (Fig. 2). Strict infection control measures were ordered by the German government on 13 March 2020, including a nationwide lockdown of schools and kindergartens. We therefore compared ED numbers in 2020 with previous years, each before and after the lockdown. Strikingly, ED numbers in 2020 before the lockdown were significantly higher than in the previous years, but significantly lower after the lockdown (Table 2).

**Table 2:** Visits at the pediatric emergency department numbers in 2020 and previous years

Time period	Weekly visits in pediatric emergency department Median [min;max]		Wilcoxon-Test
	2020	2015–2019	
Calendar week 2–12 (before lockdown)	204 [137;249]	165 [134;188]	*p* = 0.1
Calendar week 13–52 (after lockdown)	88 [64;124]	129 [98;199]	*p* < 0.01

### Pandemic action plan for respiratory infections

**Fig. 3:**
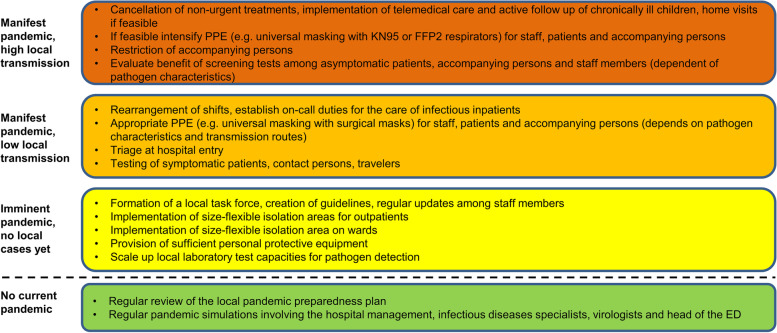
Suggested pandemic action plan for respiratory infections. *Abbreviations: ED* emergency department, *PPE* personal protective equipment

According to our observations and experience we ranked single infection control measures according to the stage of local transmission. This resulted in a pandemic action plan for respiratory infections with a step-wise approach (Fig. 3).

## Discussion

Children’s hospitals all over the world have faced particular challenges due to the novelty of SARS-CoV-2. Nosocomial spread of SARS-CoV-2 seemed to be a major threat and COVID-19-specific infection control procedures needed rapid implementation, including case definitions, screening guidelines, early triage, and patient flow across pediatric emergency departments [7–9]. At the same time, a strain of resources like face masks, SARS-CoV-2 PCR turnaround times of more than 24 h and no available vaccination hampered success of transmission mitigation strategies [7, 10].

The present study describes the main components of infection control management and infection related findings in our tertiary care children’s hospital during the COVID-19 pandemic. For the first time, we report impact of transmission mitigation strategies on non-infection related findings such as perceived effectiveness and burden of different infection control measures based on two surveys among pediatric hospital staff and caregivers.

### Infection prevention and control

The main pillars of SARS-CoV-2 infection control strategies reported from children’s hospitals were implemented in our setting and comprised nasopharyngeal/nasal screening of patients and hospital staff, implementation and adequate use of personal protective equipment (PPE), cohorting patients, limiting exposure through visitor restrictions and cancellation of non-urgent appointments, use of telemedicine options, home-office options for non-clinical work, and timely and accurate information sharing and communication [11, 12]. However, rapid upscaling of isolation areas to separate patient flows could only be implemented 2 months after the beginning of the pandemic in our setting, since the building had not been designed for the installation of isolation areas. Additional barriers of implementation included shortage of sufficient PPE, lack of staff for triage, and limited SARS-CoV-2 screening capacities hampering universal testing. As reported from other European pediatric EDs, no specific pandemic preparedness plan previously existed at our children’s hospital and no simulations for possible pandemics had been conducted beforehand [10].

Successful pandemic management in pediatric hospitals means acceptance of infection prevention and control measures by frontline workers. Acceptance has been attributed to the formation of dynamic incident management teams and timely and accurate communication [13, 14]. We believe, that the early formation of an institutional, multidisciplinary task force was the key for successful pandemic management in our Children’s Hospital. This allowed a coordinated pandemic response and timely implementation of infection control measures and counteracted confusion or misinformation generated by various streams of information within the complex structure of a tertiary care hospital.

Although not perfectly reliable, rapid antigen-tests have been recommended as a useful screening instrument for SARS-CoV-2 due to the rapid turnaround time, the simplicity and the relatively low costs [15]. The broadening of test criteria, from symptomatic travelers at the very beginning of the pandemic to standardized, universal screenings, has been reported from other children’s hospitals in Europe. This approach has been justified by the finding that transmission from asymptomatic individuals may account for more than half of all transmissions [16, 17]. Given the high number of patients at risk treated in our hospital, early detection of asymptomatic SARS-CoV-2 shedding in parents and regular screening of health-care staff is equally important [18]. Our case of a febrile patient tested positive for SARS-CoV-2 8 days after admission points at the necessity to repeatedly test patients with unexplained symptoms and those with prolonged hospitalization [19].

### Infection related findings

During the study period, a single case of in-hospital SARS-CoV-2 transmission was detected in our children’s hospital. It is impossible to compare the effectiveness of single infection control measures on nosocomial transmission. However, the introduction of a strict hygiene bundle in a similar setting as ours was associated with a significant reduction of nosocomial SARS-CoV-2 transmission [20]. Moreover, a study among 703 health care workers in a tertiary care university hospital showed that inappropriate use of personnel protective equipment while caring for patients with COVID-19 was one of the most important risk factors for infection. Other equally important risk factors were staying in the same personnel break room without a medical mask for more than 15 min, consuming food within 1 m, and failure to keep a safe social distance [21]. Collectively, these data show that strict staff adherence to interventional bundle strategies including mandatory face masks and screening for SARS-CoV-2 infections can be effective in the prevention of a high rate of nosocomial transmission or outbreaks. We emphasize that maintaining staff compliance with proper use of PPE will become even more important the longer the pandemic lasts.

### Non-infection related findings

#### Perception of infection control measures among hospital staff and caregivers

Frontline health-care workers are at increased risk of contracting SARS-CoV-2 while assessing and managing patients not only on COVID-19 wards, but also in routine care or from infected colleagues [22]. Clear infection control strategies for health-care workers have been considered essential to engender trust in the workplace [23]. For the first time, we assessed perceived effectiveness and burden of different infection control measures among staff in a tertiary care children’s hospital. Information sharing and communication about all aspects of the pandemic (e.g. regular email updates including newly developed local guidelines) received a positive feedback among 82% of staff members and probably contributed to the adoption of infection control measures. Feeling well informed and comfortable with the transmission mitigation strategy and proper use of PPE might have contributed to the perception of health-care staff of being well protected against SARS-CoV-2 infection in our setting. In contrast, in a Canadian tertiary care hospital, pediatric health-care workers were more concerned about contracting COVID-19 at work than about contracting it outside work [14]. Obligatory face masks, screening patients and caregivers and SARS-CoV-2 vaccination ranked highest in perceived effectiveness by health-care workers, whereas treatment cancellations were rated least effective. Despite being perceived as a very effective measure for infection control, half of the frontline health-care workers felt very or rather burdened by the obligation to wear face masks, in particular N95 respirators. Mandatory medical face masks in hospitals have been attributed to a decline in the incidence of hospital-acquired COVID-19 [24, 25] Thus, universal masking seems to form an important part for nosocomial transmission mitigation of respiratory infections during the present and future pandemics. In contrast, it remains controversial whether the universal use of N95 respirators (except during aerosol-generating procedures) in health-care settings additionally reduces transmission [26].

Screening of patients, caregivers and staff was considered an effective infection control measure by the vast majority of our survey participants. Of note, health-care workers experienced their own tests - if at all - less burdening compared to screening tests in caregivers or patients. Possibly, this result reflects the caring nature of health-care personnel in the pediatric setting. Despite the perceived burden, especially for pediatric patients, universal and repeated screening strategies seem to be an accepted infection control measure among hospital staff.

Strategies to limit exposure to SARS-CoV-2 within children’s hospitals include restriction of visitors and cancelling or postponing non-urgent appointments in order to prevent crowding, re-allocate consultation and ward rooms, and to preserve medical staff for care of potential COVID-19 patients [10, 27]. Almost 30% of pre-arranged outpatient appointments and more than 10% of planned inpatient appointments had to be cancelled or postponed due to the pandemic, as reported by the caregivers of sick children interviewed. Importantly, in our study, health-care staff perceived treatment cancellations as the least effective infection prevention measure combined with a high burden for sick children and their parents. Half of all health-care workers in our study experienced that care for chronically ill children had particularly suffered. Perception was probably intensified by empty consultation schedules and idle health-care staff. The lesson that can be drawn from these results is that appointment cancellations in pediatric in- and outpatient care must be weighed carefully in order to prevent deterioration of care for acutely and chronically ill children [28, 29]. If treatment cancellations are unavoidable, patients should be followed up proactively, i.e. by phone, via telemedicine tools, or by home visits.

Visitor restrictions in pediatric health-care should be kept ready as an escalation step for scenarios of high local virus transmission and adapted to the respective risk groups. Obligatory masking combined with rapid antigen tests at hospital entry are probably sufficient to prevent most hospital-acquired infections, maintain quality of routine health care for children at a high level, and allow sick children to receive visitors.

#### Maintenance of pediatric emergency care

Cancellation of routine treatments and operations in pediatrics freed human resources in our hospital, allowed the implementation of a pediatric COVID-19/respiratory infections ward and aimed to have sufficient staff available to care for children with COVID-19. However, instead of the initially expected increase, emergency outpatient visits significantly decreased in our children’s hospital compared to previous years, as already noticed by others [10]. COVID-19 patients were treated on-site with sufficient bed capacities and no overload of human resources on the infection ward and in the PICU was noted. A probable reason is a reduced transmission of respiratory infections caused by general hygiene measures and the lockdown of schools and kindergartens. While the beginning of the first wave of the pandemic in spring 2020 coincided with the end of the acute respiratory infection season at our hospital, respiratory syncytial (RS) and influenza virus infections were not detected at all during the second and third wave in winter 2020/21. Another reason that might have contributed to the low capacity utilization at our hospital is a restraint of patients seeking medical care due to anxiety of being infected by SARS-CoV-2 within the hospital [30, 31]. Our data underlines that a pandemic caused by respiratory pathogens does not necessarily lead to an increase in hospital case numbers, but can instead reduce utilization of healthcare institutions. However, this largely depends on the pathogen’s contagiousness, its disease severity and the occurrence of mutations. The present study is subject to several limitations. First, it reports on the approach of a single pediatric tertiary care hospital and a small number of children infected with SARS-CoV-2, which limits strong conclusions. Infection control strategies were adapted to local conditions and need modification in other hospital settings, countries or future pandemics with different epidemiological characteristics. Second, the participation in the survey was high but, as expected, not complete. Therefore, a potential bias is that health-care workers or caregivers who perceived infection control measures as less effective tended not to participate in the survey. However, the opposite could also be the case. Third, as only a fraction of health-care workers and no children had received SARS-CoV-2 vaccination during the study period, we could not investigate its effect on infection control. After widespread implementation of vaccination, additional studies are needed to understand the vaccination effect on infection control in pediatric hospitals. Despite these limitations, the strength of our study is the large number of participants. Our findings are based on 3716 children tested for SARS-CoV-2, 219 members of the hospital staff and 229 caregivers of different families surveyed. Most important, our study provides for the first time a real insight into the perception of infection control measures among health-care staff.

## Conclusion

In an emerging and dynamically evolving respiratory pandemic, preparedness, decisive containment action, overarching communication structures and the ability to adapt rapidly to changing challenges are critical to the management of tertiary care children’s hospitals. In summary, we show that proactive, transparent decision-making is of particular importance for hospital staff alongside effective SARS-CoV-2 transmission mitigation measures (e.g. universal masking and infection screening). However, visitor restrictions and cancellation of routine appointments were assessed as a particular burden with negative consequences for patient care and should be avoided.

We recommend that children’s hospitals, especially those with a relevant proportion of susceptible high-risk patients, have a pandemic action plan in place to avoid nosocomial transmission while maintaining adequate care for non-pandemic related sick children. We drafted a plan according to our experience and ranked single infection control measures according to the stage of local transmission. This pandemic action plan may guide children’s hospitals in the future.

## Supplementary Information

**Additional file 1.** SARS-CoV-2 diagnostic test procedures.

**Additional file 2.** Questionnaire of the online survey among health-care staff and extract of the questionnaire for caregivers (English translation).

**Additional file 3.** Results of survey on the perception of infection control measures by hospital staff, stratified by profession.

## Data Availability

The datasets used and/or analyzed during the current study are available from the corresponding author on reasonable request.

## Abbreviations

COVID-19: CoronaVirus Infectious Disease – 2019
ED: Emergency Department
FFP2: Filtering Face Piece 2
N95: N95 particulate filtering facepiece respirator
PICU: Pediatric Intensive Care Unit
PCR: Polymerase Chain Reaction
PPE: Personal Protective Equipment
