# Supplementary material for: Infection control of COVID-19 in pediatric tertiary care hospitals: challenges and implications for future pandemics
Source: BMC Pediatr. 2022 Apr 26;22:229. doi: 10.1186/s12887-022-03299-x (PMC9039594; doi:10.1186/s12887-022-03299-x)
Supplement: Supplementary file 1 — Additional file 1. SARS-CoV-2 diagnostic test procedures. [file 12887_2022_3299_MOESM1_ESM.docx]

*Table 2: SARS-CoV-2 diagnostic test procedures*

| **Implementation**  **time point** | **Test procedure for SARS-CoV-2 detection** | **Device/test name (manufacturer)** | **Sample-to-result time** | **Test location** | **Test capacity** |
| --- | --- | --- | --- | --- | --- |
| February 2020 | Real-time RT-PCR | RealStar SARS-CoV-2 assay (Altona Diagnostics) on a LightCycler 480 II device after nucleic acid extraction on a MagNA Pure 96 (Roche) | 6-8 hours | Virology laboratory | Three runs/day,  90 samples/run |
| May 2020 | Laboratory-developed RT-PCR open access protocol^4^ | Automated Panther Fusion machine (Hologic) | 5-6 hours | Virology laboratory | 300-400 samples/day |
| May 2020 | Cartridge-based RT-PCR | Xpert Xpress SARS-CoV-2 (Cepheid) on a GeneXpert instrument | 2 hours | Virology laboratory | 50-70 samples/day |
| July 2020 | TMA assay | Aptima SARS-CoV-2 TMA assay on the random-access Panther machine (Hologic) | 5-6 hours | Virology laboratory | > 500 samples/day |
| November 2020 | Cartridge-based RT-PCR (Point-of-care concept) | Xpert Xpress SARS-CoV-2 (Cepheid) on a GeneXpert instrument (only outside of lab operating hours under supervision of the virology department) | 1 hour | Pediatric ED | Up to 4 samples/hour |
| November 2020 | Rapid antigen tests | Panbio COVID-19 Ag Rapid Test Device (Abbott)  NADAL COVID-19 Ag Test (nal von minden GmbH)  SARS-CoV-2 Rapid Antigen Test (Roche) | 15-30 minutes | Pediatric ED, outpatient clinics and wards | As needed |

*Abbreviations:* *SARS-CoV-2* severe acute respiratory syndrome coronavirus 2, *RT-PCR* reverse transcription polymerase chain reaction, *TMA* transcription-mediated amplification, *ED* emergency department, *COVID-19* coronavirus disease 2019
