# Supplementary material for: Infection control of COVID-19 in pediatric tertiary care hospitals: challenges and implications for future pandemics
Source: BMC Pediatr. 2022 Apr 26;22:229. doi: 10.1186/s12887-022-03299-x (PMC9039594; doi:10.1186/s12887-022-03299-x)
Supplement: Supplementary file 2 — Additional file 2. Questionnaire of the online survey among health-care staff and extract of the questionnaire for caregivers (English translation). [file 12887_2022_3299_MOESM2_ESM.pdf]

Questionnaire for healthcare staff (translated into English)

1) Age

□ < 20 years □ 20 – 29 years □ 30 – 39 years □ 40 – 49 years □ 50 - 59 years □ ≥ 60 years □ NA

2) Gender

□ female □ male □ other

3) Profession

□ Nurse □ Physician □ Other: ________ □ NA

4) Have you been infected with COVID-19 (symptoms + positive PCR test)?

□ yes □ no □ NA

5) Have you been vaccinated against SARS-CoV-2 or are you planning to get vaccinated?

□ I have been vaccinated once or twice □ I am planning to get vaccinated
□ I am not planning to get vaccinated □ NA

6) How well have you felt protected from COVID-19 by the infection control measures at your children's hospital so far?

□ not protected □ rather not protected □ partly protected □ rather well protected □ well protected □ NA

7) How well have you been informed about the current status of the pandemic and infection control measures at the children's hospital?

□ not informed □ rather not informed □ partly informed □ rather well informed □ well informed □ NA

8) How effective in the prevention of infections among staff and patients do you rate the following measures at your children's hospital ?

A) General obligation to wear masks in the hospital
□ not effective □ rather not effective □ partly effective □ rather effective □ very effective □ NA

B) Triage at hospital entry and isolation areas for potentially contagious outpatients
□ not effective □ rather not effective □ partly effective □ rather effective □ very effective □ NA

C) Screening for SARS-CoV-2 infection by PCR at admission (patients)
□ not effective □ rather not effective □ partly effective □ rather effective □ very effective □ NA

D) Screening for SARS-CoV-2 infection by antigen-test at admission (parents/accompanying persons)
□ not effective □ rather not effective □ partly effective □ rather effective □ very effective □ NA

E) Regular screening for SARS-CoV-2 infection by antigen-test (hospital staff)
□ not effective □ rather not effective □ partly effective □ rather effective □ very effective □ NA

F) Restriction of the number of accompanying persons and visitors
□ not effective □ rather not effective □ partly effective □ rather effective □ very effective □ NA

G) Cancellation of non-urgent treatments
□ not effective □ rather not effective □ partly effective □ rather effective □ very effective □ NA

H) Restrictions of staff assemblies, trainings and business trips

□ not effective □ rather not effective □ partly effective □ rather effective □ very effective □ NA

I) Vaccination of hospital staff against SARS-CoV-2
□ not effective □ rather not effective □ partly effective □ rather effective □ very effective □ NA

9) In your perception, how burdened are patients and accompanying persons by the following measures at the children's hospital?

A) General obligation to wear masks in the hospital
□ not burdening □ rather not burdening □ partly burdening □ rather burdening □ very burdening □ NA

B) Triage at hospital entry and isolation areas for potentially contagious outpatients
□ not burdening □ rather not burdening □ partly burdening □ rather burdening □ very burdening □ NA

C) Screening for SARS-CoV-2 infection by PCR at admission (patients)
□ not burdening □ rather not burdening □ partly burdening □ rather burdening □ very burdening □ NA

D) Screening for SARS-CoV-2 infection by antigen-test at admission (parents/accompanying persons)
□ not burdening □ rather not burdening □ partly burdening □ rather burdening □ very burdening □ NA

E) Restriction of the number of accompanying persons and visitors
□ not burdening □ rather not burdening □ partly burdening □ rather burdening □ very burdening □ NA

F) Cancellation of non-urgent treatments
□ not burdening □ rather not burdening □ partly burdening □ rather burdening □ very burdening □ NA

10) How burdened do you feel by the by the following measures at the children's hospital?

A) General obligation to wear masks in the hospital
□ not burdening □ rather not burdening □ partly burdening □ rather burdening □ very burdening □ NA

B) Regular screening for SARS-CoV-2 infection by antigen-test (hospital staff)
□ not burdening □ rather not burdening □ partly burdening □ rather burdening □ very burdening □ NA

J) Restrictions of staff assemblies, trainings and business trips
□ not burdening □ rather not burdening □ partly burdening □ rather burdening □ very burdening □ NA

K) Vaccination of hospital staff against SARS-CoV-2
□ not burdening □ rather not burdening □ partly burdening □ rather burdening □ very burdening □ NA

11) How much did the maintenance of care for chronically ill children suffer by infection control measures at the children's hospital during the pandemic?
□ it did not suffer □ it did rather not suffer □ it suffered in parts □ it rather suffered □ it suffered □ NA

12) Have you experienced complications caused by the infection control measures (e.g. delay of an urgently needed treatment

□ no □ yes, sporadically □ yes, frequently □ NA

13) Do you want to tell us something?

Extract of questionnaire for caregivers of pediatric outpatients (translated into English)

The following questions refer to the entire duration of the Coronavirus pandemic:

1) How high do you rate the risk, that your child(ren) get infected with the Coronavirus during everyday-life situations (e.g. in the supermarket, on the playground)?

   □ Very high □ high □ moderate □ small □ no risk □ NA

2) How high do you rate the risk, that your child(ren) get infected with the Coronavirus during a visit at your local pediatrician or general practitioner?

   □ Very high □ high □ moderate □ small □ no risk □ NA

3) How high do you rate the risk, that your child(ren) get infected with the Coronavirus during a visit at the children's hospital?

   □ Very high □ high □ moderate □ small □ no risk □ NA
