# Supplementary material for: Infection control of COVID-19 in pediatric tertiary care hospitals: challenges and implications for future pandemics
Source: BMC Pediatr. 2022 Apr 26;22:229. doi: 10.1186/s12887-022-03299-x (PMC9039594; doi:10.1186/s12887-022-03299-x)
Supplement: Supplementary file 3 — Additional file 3. Results of survey on the perception of infection control measures by hospital staff, stratified by profession. [file 12887_2022_3299_MOESM3_ESM.pdf]

Results of survey on the perception of infection control measures by hospital staff, stratified by profession

How effective in the prevention of infections among staff and patients do you rate the following measures at your children's hospital ?

	total		nurses		physicians		other	
	n	%	n	%	n	%	n	%
General obligation to wear masks in the hospital								
very effective	154	70%	79	72%	31	79%	38	60%
rather effective	51	23%	23	21%	6	15%	21	33%
partly effective	10	5%	6	5%	1	3%	3	5%
rather ineffective	2	1%	1	1%	1	3%	0	0%
ineffective	1	0%	0	0%	0	0%	1	2%
NA	1	0%	1	1%	0	0%	0	0%
Triage at hospital entry and isolation areas for potentially contagious outpatients								
very effective	101	46%	52	47%	17	44%	28	44%
rather effective	68	31%	27	25%	16	41%	22	35%
partly effective	37	17%	23	21%	4	10%	10	16%
rather ineffective	7	3%	4	4%	2	5%	1	2%
ineffective	1	0%	0	0%	0	0%	1	2%
NA	5	2%	4	4%	0	0%	1	2%
Screening for SARS-CoV-2 infection by PCR at admission (patients)								
very effective	153	70%	83	75%	24	62%	39	62%
rather effective	49	22%	21	19%	12	31%	16	25%
partly effective	9	4%	3	3%	3	8%	3	5%
rather ineffective	2	1%	1	1%	0	0%	1	2%
ineffective	1	0%	0	0%	0	0%	1	2%
NA	5	2%	2	2%	0	0%	3	5%
Screening for SARS-CoV-2 infection by antigen-test at admission (parents/accompanying persons)								
very effective	154	70%	87	79%	22	56%	38	60%
rather effective	47	21%	16	15%	14	36%	17	27%
partly effective	9	4%	3	3%	2	5%	4	6%
rather ineffective	3	1%	1	1%	1	3%	1	2%
ineffective	1	0%	0	0%	0	0%	1	2%
NA	5	2%	3	3%	0	0%	2	3%
Regular screening for SARS-CoV-2 infection by antigen-test (hospital staff)								
very effective	114	52%	62	56%	13	33%	35	56%
rather effective	57	26%	23	21%	14	36%	18	29%
partly effective	32	15%	17	15%	10	26%	5	8%
rather ineffective	7	3%	4	4%	2	5%	1	2%
ineffective	3	1%	2	2%	0	0%	1	2%
NA	6	3%	2	2%	0	0%	3	5%
Restriction of the number of accompanying persons and visitors								
very effective	141	64%	73	66%	25	64%	39	62%
rather effective	42	19%	19	17%	9	23%	12	19%
partly effective	31	14%	15	14%	5	13%	10	16%
rather ineffective	4	2%	3	3%	0	0%	1	2%
ineffective	1	0%	0	0%	0	0%	1	2%
NA	0	0%	0	0%	0	0%	0	0%

Cancellation of non-urgent treaments								
very effective	59	27%	28	25%	10	26%	18	29%
rather effective	60	27%	26	24%	13	33%	21	33%
partly effective	60	27%	37	34%	8	21%	13	21%
rather ineffective	23	11%	12	11%	6	15%	4	6%
ineffective	8	4%	3	3%	2	5%	2	3%
NA	9	4%	4	4%	0	0%	5	8%
Restrictions of staff assemblies, trainings and business trips								
very effective	100	46%	47	43%	15	38%	33	52%
rather effective	65	30%	34	31%	10	26%	19	30%
partly effective	41	19%	22	20%	12	31%	7	11%
rather ineffective	6	3%	3	3%	1	3%	2	3%
ineffective	1	0%	0	0%	0	0%	1	2%
NA	6	3%	4	4%	1	3%	1	2%
Vaccination of hospital staff against SARS-CoV-2								
very effective	150	68%	68	62%	34	87%	43	68%
rather effective	31	14%	21	19%	1	3%	8	13%
partly effective	17	8%	13	12%	1	3%	3	5%
rather ineffective	5	2%	3	3%	1	3%	1	2%
ineffective	3	1%	1	1%	0	0%	2	3%
NA	13	6%	4	4%	2	5%	6	10%

In your perception, how burdened are patients and accompanying persons by the following measures at the children's hospital?

	**total**		**nurses**		**physicians**		**other**	
	**n**	**%**	**n**	**%**	**n**	**%**	**n**	**%**
General obligation to wear masks in the hospital								
very burdened	30	14%	21	19%	5	13%	4	6%
rather burdened	66	30%	35	32%	12	31%	19	30%
partly burdened	56	26%	23	21%	11	28%	19	30%
rather not burdened	39	18%	16	15%	7	18%	13	21%
not burdened	20	9%	10	9%	4	10%	6	10%
NA	8	4%	5	5%	0	0%	2	3%
Triage at hospital entry and isolation areas for potentially contagious outpatients								
very burdened	11	5%	6	5%	0	0%	5	8%
rather burdened	43	20%	25	23%	4	10%	14	22%
partly burdened	51	23%	22	20%	12	31%	14	22%
rather not burdened	73	33%	33	30%	17	44%	20	32%
not burdened	31	14%	19	17%	5	13%	7	11%
NA	10	5%	5	5%	1	3%	3	5%
Screening for SARS-CoV-2 infection by PCR at admission (patients)								
very burdened	21	10%	15	14%	4	10%	2	3%
rather burdened	50	23%	24	22%	13	33%	11	17%
partly burdened	59	27%	30	27%	8	21%	20	32%
rather not burdened	57	26%	26	24%	9	23%	19	30%
not burdened	20	9%	8	7%	4	10%	8	13%
NA	12	5%	7	6%	1	3%	3	5%

Screening for SARS-CoV-2 infection by antigen-test at admission (parents/accompanying persons)								
very burdened	15	7%	11	10%	1	3%	3	5%
rather burdened	40	18%	21	19%	7	18%	8	13%
partly burdened	61	28%	27	25%	15	38%	19	30%
rather not burdened	62	28%	33	30%	10	26%	17	27%
not burdened	30	14%	14	13%	5	13%	11	17%
NA	11	5%	4	4%	1	3%	5	8%
Restriction of the number of accompanying persons and visitors								
very burdened	105	48%	57	52%	18	46%	27	43%
rather burdened	64	29%	28	25%	15	38%	19	30%
partly burdened	23	11%	10	9%	4	10%	8	13%
rather not burdened	8	4%	4	4%	1	3%	3	5%
not burdened	9	4%	6	5%	1	3%	2	3%
NA	10	5%	5	5%	0	0%	4	6%
Cancellation of non-urgent treaments								
very burdened	65	30%	31	28%	14	36%	18	29%
rather burdened	74	34%	33	30%	18	46%	20	32%
partly burdened	43	20%	25	23%	3	8%	14	22%
rather not burdened	10	5%	5	5%	3	8%	2	3%
not burdened	9	4%	6	5%	0	0%	3	5%
NA	18	8%	10	9%	1	3%	6	10%

How burdened do you feel by the by the following measures at the children's hospital?

	**total**		**nurses**		**physicians**		**other**	
	**n**	**%**	**n**	**%**	**n**	**%**	**n**	**%**
General obligation to wear masks in the hospital								
very burdened	48	22%	34	31%	6	15%	7	11%
rather burdened	60	27%	38	35%	7	18%	15	24%
partly burdened	42	19%	18	16%	9	23%	15	24%
rather not burdened	31	14%	6	5%	7	18%	14	22%
not burdened	35	16%	13	12%	9	23%	11	17%
NA	3	1%	1	1%	1	3%	1	2%
Regular screening for SARS-CoV-2 infection by antigen-test (hospital staff)								
very burdened	4	2%	3	3%	0	0%	1	2%
rather burdened	8	4%	6	5%	1	3%	1	2%
partly burdened	29	13%	20	18%	3	8%	5	8%
rather not burdened	74	34%	45	41%	15	38%	12	19%
not burdened	87	40%	32	29%	18	46%	33	52%
NA	17	8%	4	4%	2	5%	11	17%
Restrictions of staff assemblies, trainings and business trips								
very burdened	7	3%	2	2%	2	5%	3	5%
rather burdened	35	16%	16	15%	10	26%	8	13%
partly burdened	58	26%	31	28%	13	33%	14	22%
rather not burdened	62	28%	40	36%	7	18%	15	24%
not burdened	53	24%	20	18%	6	15%	21	33%
NA	4	2%	1	1%	1	3%	2	3%

Vaccination of hospital staff against SARS-CoV-2								
very burdened	9	4%	5	5%	1	3%	3	5%
rather burdened	6	3%	4	4%	0	0%	0	0%
partly burdened	16	7%	11	10%	0	0%	5	8%
rather not burdened	49	22%	24	22%	9	23%	16	25%
not burdened	119	54%	57	52%	26	67%	31	49%
NA	20	9%	9	8%	3	8%	8	13%
